# Negative Emotion under Haze: An Investigation Based on the Microblog and Weather Records of Tianjin, China

**DOI:** 10.3390/ijerph16010086

**Published:** 2018-12-30

**Authors:** Xuan Sun, Wenting Yang, Tao Sun, Ya Ping Wang

**Affiliations:** 1 Zhou Enlai School of Government, Nankai University, Tianjin 300350, China; sunxuan@nankai.edu.cn; 2 Experimental Teaching Center of Applied Social Science, Nankai University, Tianjin 300350, China; 3 School of International and Public Affairs, Shanghai Jiao Tong University, Shanghai 200240, China; sjtuywt@sjtu.edu.cn; 4 School of Social & Political Sciences, University of Glasgow, Glasgow G12 8RS, UK; yaping.wang@glasgow.ac.uk

**Keywords:** public emotion, haze, microblog, mental health, China

## Abstract

Nowadays, many big cities are suffering from heavy air pollution and continuous haze weather. Compared with the threat on physical health, the influence of haze on people’s mental health is much less discussed in the current literature. Emotion is one of the most important indicators of mental health. To understand the negative impact of haze weather on the emotion of the people, we conducted an investigation based on historical weather records and microblog data in Tianjin, China. Specifically, an emotional thesaurus was generated with a microblog corpus collected from sample data. Based on the thesaurus, the public emotion under haze was statistically described. Then, through correlation analysis and comparative study, the relation and seasonal variation of haze and negative emotion of the public were well discussed. According to the study results, there was indeed a correlation between haze and negative emotion of the public, but the strength of this relationship varied under different conditions. The level of air pollution and weather context were both important factors that influence the mental effects of haze, and diverse patterns of negative emotion expression were demonstrated in different seasons of a year. Finally, for the benefit of people’s mental health under haze, recommendations were given for haze control from the side of government.

## 1. Introduction

Along with economic development and natural resource exploitations, many environmental problems have arisen around the world and to a different extent have influenced the daily lives of people [1,2]. Due to fast, large-scale industrialization and the extensive use of fossil fuels [3,4], haze has become one of the most common-seen climate phenomena in many big cities [5]. Heavy air pollution of the haze weather poses a great risk to the public health [6,7], especially in the South and Southeast Asian countries, such as China, Malaysia, Indonesia, and even Singapore, the air quality in urban areas is often very poor nowadays [8,9,10,11].

A great deal of research work has been done to examine the impacts of haze on the physical health of the public. According to the medical statistics, the morbidity of cardiovascular, cerebrovascular, and respiratory diseases is closely associated with haze [12], and hospital admissions increase significantly in haze weather [13]. Meanwhile, there are also strong evidences showing that haze is a potentially modifiable risk factor for lung cancer [14] and an underlining cause for the high mortality in some places [15]. To better understand the effect mechanism, the chemical composition of air pollutants and their sources, regional flows, and interactions have been widely studied [16,17,18]. On the basis of these studies, a number of protection measures [19,20] and policies [21,22,23] were proposed out to reduce the influences of the haze from different perspectives.

However, the threat of the haze is not just to people’s physical health but also to their mental health. From the perspective of the environmental psychology, the mentality of individuals is largely determined by the built and natural environments [24]. Yet, the effect of haze on mental health has not been widely discussed within the current literature. With the course of urbanization, mental problems among the population have become increasingly prevalent and serious in recent years [25]. Although it is proven that the improvement of living conditions [26], such as increasing green and blue spaces in the neighborhood [27,28], is beneficial to mental health, the challenges from climate change are still non-negligible [29,30,31]. Emotion is one of the most important indicators of mental health. As demonstrated in previous studies, daily moods and sentiment are potentially affected by the weather and reflected in outward expressions and behaviors [32,33]. With the online diary and weather station data, Denissen et al. [34] studied the mental effects of temperature, wind power, sunlight, precipitation, air pressure, and photoperiod. Using an experience-sampling method, Kööts et al. [35] examined the relationship between affective experiences and weather variables. Lucas et al. [36] discussed the association between daily weather conditions and life satisfaction of American people through a cross-sectional investigation. As for the effect of haze, Zhang et al. [37] conducted a nationwide longitudinal survey in China, revealing that a dirty sky did have some negative impacts on mental health and the subjective well-being of people.

In today’s highly information-based society, an increasing number of people prefer to express their opinions and feelings on the web, particularly through the social media [38]. Judging from the verbal expression and reactions of people in the digital world, their cognition, likes, and dislikes can be to some extent depicted [39]. Thus, many works have been done to study the emotion and sentiments of the public based on social media data [40,41,42]. With the results of sentiment analysis, it is possible to improve our knowledges of citizens’ political preferences [43], stock movements [44], trends of financial markets [45], and so on. Meanwhile, to figure out the psychological effects of terrors [46] and various stressful events that people may experience [47], the emotions expressed in the social media are also significant clues.

Based on the microblog and weather records of Tianjin, China, we investigate the relation between haze weather and negative emotion of the public in this paper. Through historical data analysis, not only was the correlation between the two proven, but the seasonal variation was also discussed. The contributions of the study mainly lie in the following three aspects: first, on the theoretical level, the negative impacts of haze on the mental health was explored; second, technically, the tendency of public emotion under haze weather was judged through the semantic parsing and statistical analysis of microblog data; and third, according to the investigation results, some suggestions are given for haze control from the perspective of mental health protection.

## 2. Methodology

### 2.1. Study Scope and Research Design

**Figure 1:**
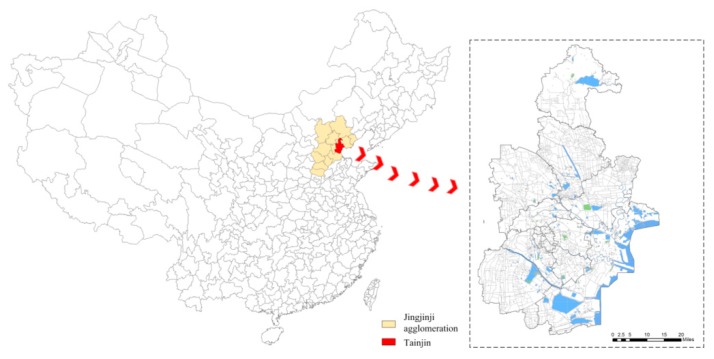
The administrative area of Tianjin in the Jingjinji agglomeration of China.

**Figure 2:**
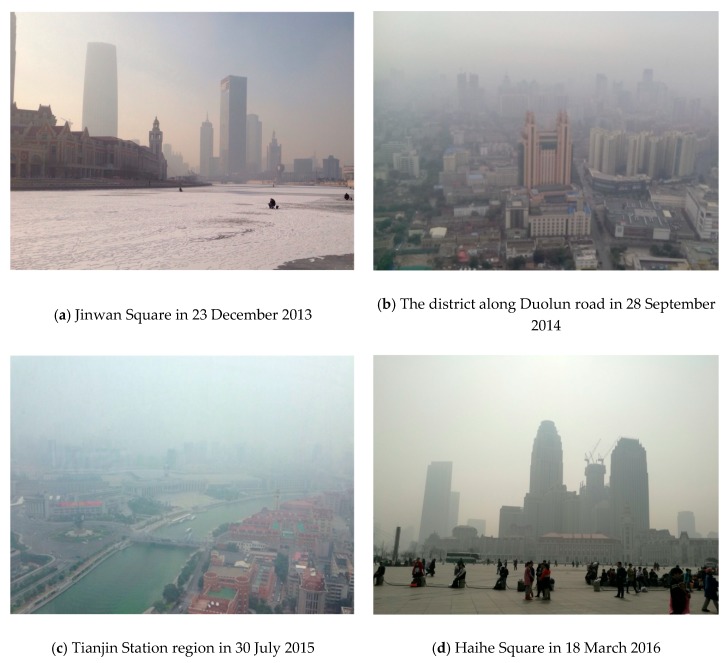
Tianjin under the haze in recent years (all the photos are taken from the posted Sina microblog messages [48]).

According to the statistics produced by the China National Environmental Monitoring Centre, from 2013 to 2017, 74 major cities were under the haze in over 100 days of a year. Compared with other places suffering from heavy air pollution, the region of Jingjinji in the northern China was the hardest-hit area. In geography, the Jingjinji region covers 2 municipalities and 11 prefecture-level cities, with a total area of 218 thousand square kilometers. Within the region accounting for about 2.35% area of China, there are about 110 million permanent residents, ~8.1% population of the country, contributing to ~10% of Chinese gross domestic product (GDP). Aside from Beijing, Tianjin is one of the central cities in the Jingjinji urban agglomeration (see Figure 1). It is not only an epitome of Chinese urbanization progress, but also a typical city that frequently experienced large-scale and continuous haze in recent years (see Figure 2). In 2014, Tianjin was ever covered by haze for about 200 days in total, and in 8 instances, the period of haze weather lasted for more than one week. The life of the people was greatly influenced by the haze, and the poor air quality was hotly discussed on the web.

In our research, Tianjin was chosen as the case study area, and the public emotion under the haze in 2014 were specifically investigated. To ensure the representativeness of the research work, the data was collected in January, April, August, and November, which were typical samples of the four different seasons of the year. On one hand, the weather records of Tianjin in the selected months were checked to decide the levels of air pollution each day; and on the other hand, within the corresponding periods of time, all the microblog messages that had mentioned “haze”, “fog”, or “bad air quality” were examined for investigation of the public’s emotions.

- Since fine particulate matter (PM) is the main cause of haze in Tianjin, as in most other Chinese cities, the average density of PM2.5 was taken as the direct indicator of air quality in this paper.
- Comparing with the microblog platforms of Tencent, Netease, and Sohu, the Sina microblog was the most active and popular one, and so it was chosen as the data source for sentiment analysis.

**Figure 3:**
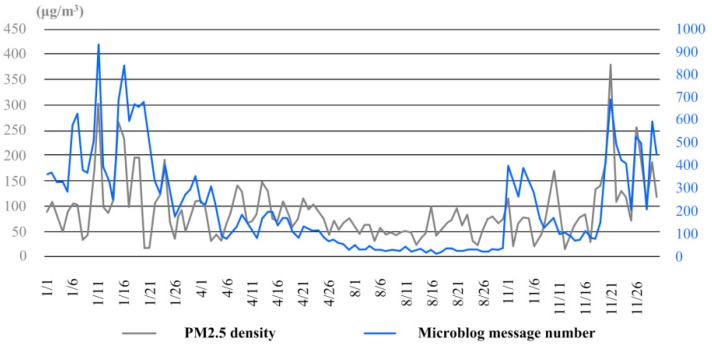
The variation curves of PM2.5 density and microblog message number in the four sample months.

Through time series analysis of both the number of Sina microblog messages concerning haze and the PM2.5 density in the air, similar variation trends were actually demonstrated, especially in the late autumn and winter (see Figure 3). For the 4 sample months, the correlation coefficient of the two factors was 0.641, showing statistically significance under the level of 0.05 on both sides.

### 2.2. Emotional Thesaurus Generation with a Microblog Corpus

As the basic unit of language, words are fundamental to emotion expression and understanding [49,50,51]. Based on the 65 emotional words of the Profile of Mood States (POMS) in psychology, Pepe and Bollen had ever developed a thesaurus with 793 words to extract mood indicators from emails [52]. With the help of OpinionFinder and Google Profile of Mood States (GPOMS), Bollen searched and selected 964 emotional words from the web to infer the public moods with Twitter [53]. Zhao et al. had mapped 95 emoticons into four categories of sentiments and developed the first sentiment analysis system for Chinese microblog [54].

With the purpose to analyze public emotion under the haze, a specific thesaurus was firstly generated with an actual microblog corpus.

#### 2.2.1. Microblog Corpus Collection

**Figure 4:**
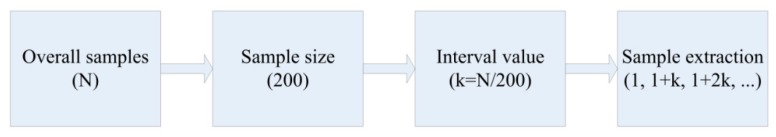
The process of K-sampling for microblog corpus collection.

When people talk about the same topic in microblog, their emotion expressions are often similar [55]. To figure out the most frequently used emotional words towards haze, the corpus was collected with the K-sampling method, which chose 4 short periods of time from different seasons in 2014 and extracted 200 sample microblog messages in each period with an uniform interval (see Figure 4). In the process of corpus collection, different lengths of sample periods were actually set to ensure the approximately equal quantity of overall samples in different seasons (see Table 1). As a result, 800 microblog messages with 45,595 words were selected as the corpus. The corpus might not cover all the possible emotional words of individuals, but it was representative of the emotion expression of the majority people under haze.

#### 2.2.2. Emotional Thesaurus Generation

Based on the microblog corpus, an emotional thesaurus was generated by NLPIR (ICTCLAS 2014), which is a word segmentation system developed by the Chinese Academy of Science [56]. It is capable of word splitting, speech discrimination, keyword extraction, etc., and there are more than 300,000 users all over the world, including some respectable research institutions, such as Qinghua University and MIT.

**Figure 5:**
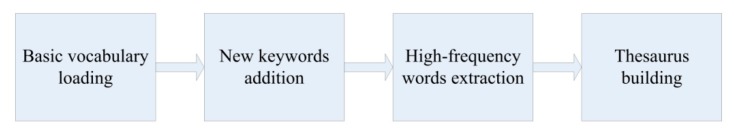
The process of emotional thesaurus generation with NLPIR.

Using the NLPIR, the process of emotional thesaurus generation can be divided into four steps (see Figure 5): first, the basic vocabulary of the NLPIR system was loaded in advance; second, some new keywords related with haze were added into the user dictionary of the NLPIR; third, based on the basic vocabulary and the new keywords, the NLPIR searched the corpus and extracted all the high-frequency words from the microblog messages concerning haze; and fourth, with reference to the meanings and parts of speech of the extracted words, proper ones were selected to build the thesaurus.

To enhance the completeness and reliability of the result, four rounds of keyword addition and word extraction were actually conducted in the whole process of thesaurus generation. Every time that the microblog corpus was analyzed with the NLPIR, some new keywords would be recommended. Following the recommendations of the system, the keywords in the user dictionary were progressively enriched (see Table 2), and in the end, 72 words were successfully extracted from the microblog messages concerning haze (see Table 3). 

Eliminating the unrelated words, as well as the nouns and verbs which cannot reflect the emotions of the people, 25 effective words were screened out from the 72 ones extracted by the NLPIR. Based on the model of positive and negative affect (PANA) proposed by Watson and Tellegen [57], the 25 effective words were further classified into two categories, the negative words and the positive words. All together, they constituted the emotional thesaurus of microblog under the haze (see Table 4).

### 2.3. Statistical Description of Public Emotion Under the Haze

Taking the emotional words, together with the keyword “haze”, as the searching words, the number of microblog messages expressing different sentiments in each day of the sample period was able to be recorded. With reference to the records of the words in the thesaurus, the public emotion under the haze were statistically described with the following function.

(1) $$EI_{s,t}=\sum_{i=1}^{N_{s}}rec_{s,t}(i),$$

where, *EI_s,t_* stands for the emotion index of certain sentiment *s* in the day of *t*, *N_s_* is the number of words that are able to express the *s* sentiment in the thesaurus, and *rec_s,t_*(*i*) is the record of microblog message number for the No. *i* word expressing the *s* sentiment in the day of *t*.

**Figure 6:**
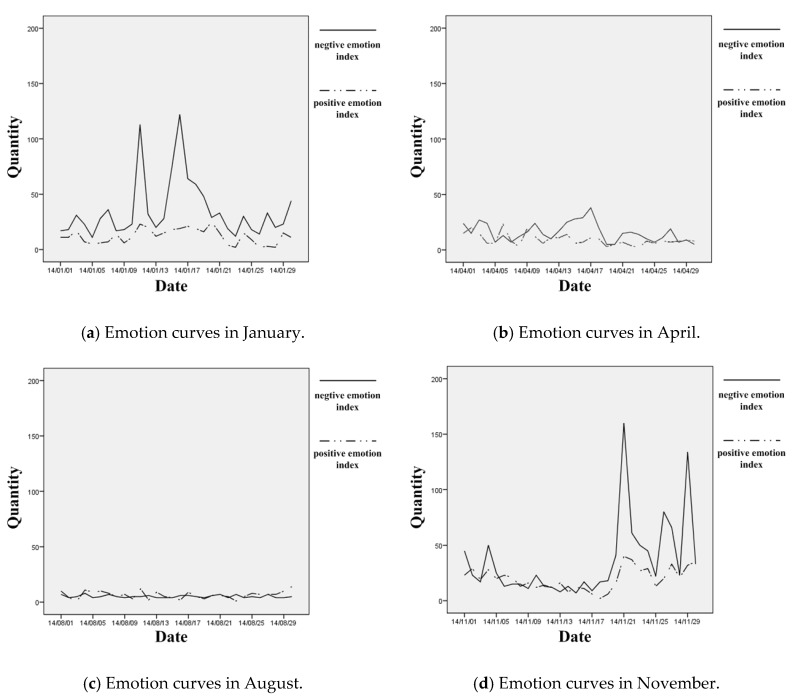
The positive and negative emotion curves in the four sample months.

Specifically, two emotion indexes were calculated based on the nine positive words and 16 negative words. Judging from the quantity-time curves of the two indexes in the four sample months (see Figure 6), the positive emotion was always at low level when it came to haze, yet the negative emotion was at the higher level for most of the time, especially in the late autumn (November) and winter (January) seasons.

In terms of the statistics (see Table 5), the overall mean value (21.61), and standard deviation (26.627) of the negative emotion index were all much higher than those of the positive emotion index (11.90, 8.274), indicating frequent expression and great fluctuation of negative emotion under the haze. While the emotion fluctuation (9.890 for the positive emotion and 36.326 for the negative emotion) was the greatest in November, the difference of the different emotion indexes was the biggest (22.47 for the mean difference) in January. In August, the summer time, both the mean values and the standard deviations of positive (6.50, 3.309) and negative indexes (2.93, 1.799) declined to the lowest, and the positive emotion expression had only the change to surpass that of the negative emotion.

## 3. Results

### 3.1. Relationship between Haze and the Negative Emotion of the Public

To study the relationship between haze and the negative emotion of the public, correlation analysis was conducted based on the PM2.5 density records and the negative emotion index calculated in each day of the sample months.

Overall, the correlation coefficient of the two factors reached up to 0.781, statistically significant under the level of 0.05 on both sides. However, the psychological influence is always a complicated mechanism. For better understanding of the effect manner of haze, we made further detailed discussions from different perspectives.

#### 3.1.1. Discussions on the Relationship at Different Haze Levels

**Figure 7:**
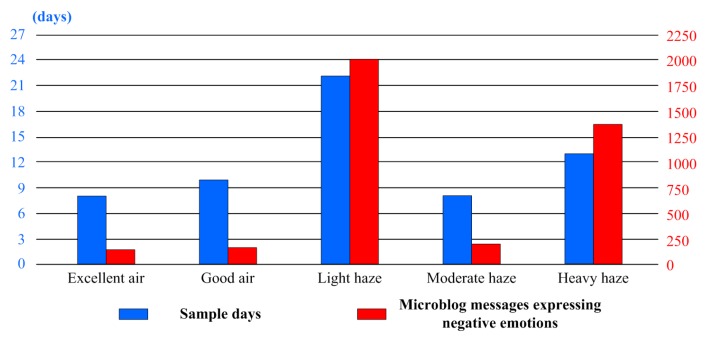
The distribution histogram of sample days and microblog messages expressing negative emotion in January and November.

According to the grading standards of air quality in China, the weather condition was generally categorized into five levels based on the PM2.5 density, from excellent air (0–35 μg/m^3^), good air (35–75 μg/m^3^), light haze (75–115 μg/m^3^), moderate haze (115–150 μg/m^3^), to heavy haze (>150 μg/m^3^). With consideration of the diversity of samples, the recorded data from January and November was sorted to discuss the relationship of haze and the negative emotion of the public at different haze levels (see Figure 7). 

Judging from the statistics and correlation analysis results of the sample data (see Table 6), the relation strength of haze and negative emotion of the public was variable under different conditions. Generally, the higher the PM2.5 density was, the closer the relationship of them could be. However, it was not a linear process. On one hand, comparing to the situation under excellent air and good air conditions, the relationship was particularly strong under light haze and heavy haze. For example, when the average PM2.5 density was approximately 95.95 and 226.54, the correlation coefficient reached as high as 0.750 and 0.829, respectively. But on the other hand, the negative emotion was not highly expressed under moderate haze. When the average PM2.5 density was ~124.67, the correlation coefficient of PM2.5 density and negative emotion index was only 0.281, even less than that under good air conditions.

#### 3.1.2. Discussions on the Relationship at Different Haze Levels

**Figure 8:**
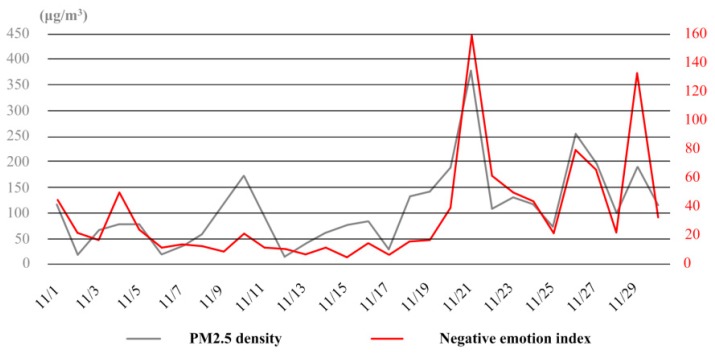
The variation curves of PM2.5 density and negative emotion index in November.

For a deeper exploration of the relationship between haze and negative emotion of the public, discussions were then directed at their temporal sequence association. With reference to the variations of air quality and public emotion, four short periods were selected out from November, which was a typical span of time with frequent haze weather and fluctuating emotion of the public (see Figure 8). Each of the periods contained six sample days. 

By comparing the statistics and correlation analysis results of the four periods (see Table 7), it was found that the mental influence of haze could hardly be assessed just with the physical parameters of air quality. For example, the air quality of the first and second periods were almost at the same level, but more negative emotion was in fact expressed in the former period. Meanwhile, the values of negative emotion indexes of the third and fourth periods were very close, yet the PM2.5 density of the third was obviously higher than the fourth one.

Thus, attention should also be given to other factors, especially the weather context, when discussing the mental effects of haze in practice. As indicated by the results of the first two sample periods in November, people reacted badly to light haze at the beginning, but when the air pollution was not so serious and the influence was limited, the negative emotion tended to reduce with the continuation of haze weather. On the contrary, when the heavy haze continued for quite a few days, just like the situation in the last two periods, the negative emotion was more likely to get enhanced.

### 3.2. Seasonal Variation of Haze and Negative Emotion of the Public

**Figure 9:**
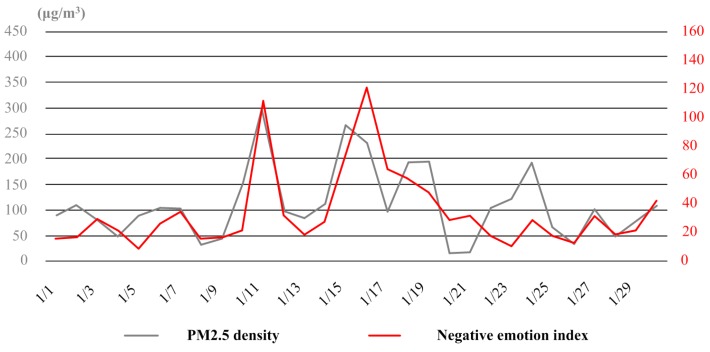
The variation curves of PM2.5 density and negative emotion index in January.

Similar to the case of November, there were also several times of heavy and continuous haze weather in January. With the fluctuation of air quality, the negative emotion was expressed to different degrees during the month (see Figure 9). When the PM2.5 density was over 200 μg/m^3^ (e.g., the periods from the date of 11st to 13th and from 15th to 17th), the response of people was immediate and emotional. However, for the days with light haze (e.g., the periods from the date of 1st to 5th and from 5th to 9th) and moderate haze (e.g., the period from the date of 23st to 25th), the response was lagged and depressed.

By contrast, much less haze had appeared in the spring and summer time, and accordingly, the amount of negative emotion expressed through the microblog declined obviously.

**Figure 10:**
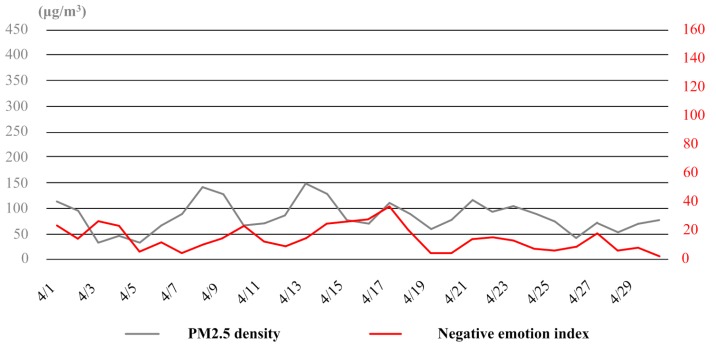
The variation curves of PM2.5 density and negative emotion index in April.

**Figure 11:**
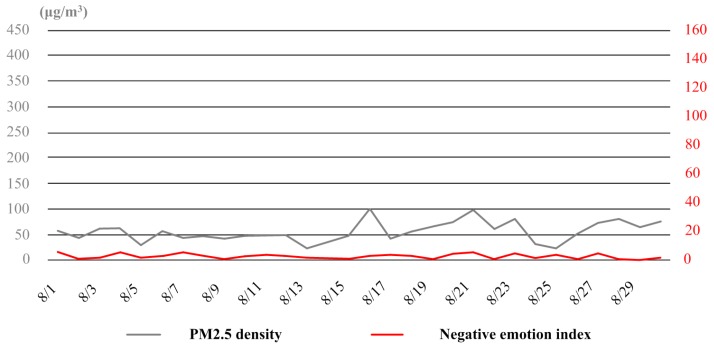
The variation curves of PM2.5 density and negative emotion index in August.

- In April, there was actually no heavy haze in Tianjin. Although sometimes the air quality was bad (e.g., the dates of 8th and 13th), for most of the time, the PM2.5 density stayed between 50 μg/m^3^ and 100 μg/m^3^. Under these weather conditions, the relationship between haze and negative emotion of the public was also detectable, but the fluctuation trends of air quality and public emotion was not as consistent as those in January and November any more (see Figure 10).
- In August, over 80% of the time Tianjin were under good and excellent air condition. Only in very few days (e.g., the dates of 16th and 21th), the PM2.5 density reached up to 100 μg/m^3^. Meanwhile, there was hardly any negative emotion expressed in the whole month. The correlation between haze and negative emotion of the public became quite weak (see Figure 11).

## 4. Conclusions and Recommendations

Due to natural and human causes, haze has become a commonly seen climate phenomenon in a lot of cities. Aside from the threat to people’s physical health, the impact of haze on mental health should never be ignored. Taking Tianjin City as a study case, we examined the negative emotion of the public under haze with historical weather records and microblog data. Based on the emotional thesaurus generated with an actual microblog corpus, the public emotion under haze was statistically described. Furthermore, through correlation analysis and comparative study, the relation and seasonal variation of haze and negative emotion of the public were well discussed.

According to the study results, there was indeed a correlation between haze and negative emotion of the public. However, the strength of this relationship varied under different conditions. We found that the level of air pollution and local weather context were both important factors that influence the mental effects of haze in practice. At the same time, due to variation in frequency and intensity of haze weather, diverse patterns of negative emotion expression could be observed in different seasons of a year. As for Tianjin, specifically, (1) while negative emotion of the public was easy to be brought about by haze in late autumn and winter, the influence of haze was not so prominent in spring and summer; (2) both light haze and heavy haze had obvious negative impacts on public emotions, and comparing to the effect of the former, the latter was always stronger; (3) the negative emotion of the public was prone to get reduced with the duration of light or moderate haze weather, but when facing severe air pollution, negative emotion was more likely to get enhanced with time.

Thus, for the benefit of the people’s mental health, it would be necessary for the government to take the negative emotion of the public into consideration in the whole work of haze control.

First, based on the microblog or some other social media platforms, a long-term monitoring and alert system needs to be established to master the dynamic change of the public emotion under haze. As the mental effects of haze could vary in different seasons, at different periods, and under different weather conditions, to respond to the various degrees of severity of threat to mental health; control measures ought to be taken according to the real-time status of public moods under haze, aside from the actual levels of air pollution.

Second, in view of the combined effects of haze on body and mind and the timing and strength of public intervention (e.g., emission restriction, air purification, and outdoor activity reduction) haze control measures need to be reconsidered. People might feel very bad under light haze, but at other points in time, hold lesser negative emotions under even heavy haze. The adoption of intervention measures should be based on the comprehensive assessment of the physical and psychological influence of haze, rather than just from one perspective.

Third, with reference to the rules of emotion change and psychotherapy theories, some new sorts of measures could be introduced to reduce the mental harm of haze. For example, through the guidance of public opinion, the focus of the people could be transferred from haze to other positive topics, especially when the negative emotion of the public is about to reach its peak. Extensive developments of indoor activities would always help to ease the frustration and impatience of the people in their spare time. In addition, the virtual reality and online community also show great potential to enrich the mental life of the people under haze.

## Figures and Tables

**Table 1:** Sampling parameters for corpus collection in different seasons.

Seasons	Sample Periods	Overall Samples (N)	Interval Value (K)
Winter	15 January 2014–20 January 2014	12,372	61
Spring	04 April 2014–15 April 2014	12,070	60
Summer	10 July 2014–07 August 2014	12,483	62
Winter	02 November 2014–November 2014	12,483	63

**Table 2:** The lists of keywords for addition in the four rounds of corpus analysis.

Rounds	Keyword Lists
1	haze, complaints, negative energy, fresh air, miserable, automobile exhaust, gas emission, traffic restriction, APEC blue, pollution control, crazy, serious haze, bad weather, very serious, haze control, sorrow, bad mood, big wind
2	northwest wind, heavy, disgusting, nausea, depressed, hate haze, heart broken
3	haze subsidies, haze reduction, enduring haze
4	air pollution, blowing wind

Note: all the keywords are translated from Chinese, and it is the same for the words in the following tables.

**Table 3:** The high-frequency words extracted by the NLPIR.

Frequencies	Word Lists
≥100	haze, weather, Tianjin
50~99	Beijing, pollution, air, sunshine
20~49	breath, serious, mood, feeling, hope, blue sky and while cloud, air quality, blowing wind, environment, gutter oil, traffic restriction, mask, expert
10~19	blue sky, sky, governance, continue, like, away, blowing big wind, damn, big wind, serious haze, PM2.5, cloudy, haze weather, grey, good weather, beautiful
5~9	covered by haze, bothering, heating supply, indulge, bad, gloom, haze subsidies, enjoy, crazy, serious pollution, great pollution, tolerate, thanks, complaint, heavy haze, horrible, waste
<5	haze control, happy, terrible, nima, cleaning the lung, smoke, helpless, sentiment, end of the world, comfortable, dispersing, fireworks, uncomfortable, tired, sorrow, not bad, hard, bright, hurt

**Table 4:** The emotional thesaurus of microblog under the haze.

Categories	Word Lists
Positive	like, good weather, beautiful, enjoy, thanks, happy, comfortable, not bad, bright
Negative	serious, away, damn, bothering, bad, gloom, tolerate, complaint, horrible, terrible, nima, helpless, end of the world, uncomfortable, tired, sorrow, hurt

**Table 5:** Statistics of the emotion indexes in the four sample months.

Months	The Minimum		The Maximum		Mean		Std. Dev.	
	Positive	Negative	Positive	Negative	Positive	Negative	Positive	Negative
January	2	9	25	122	12.03	34.50	6.641	27.361
April	2	2	23	37	9.27	14.40	5.112	8.767
August	1	0	14	6	6.50	2.93	3.309	1.799
November	2	5	40	160	19.80	34.60	9.890	36.326
Overall	1	0	40	160	11.90	21.61	8.274	26.627

**Table 6:** Statistics and correlation analysis results of the sample data at different haze levels in January and November.

Weather Conditions	Average PM2.5 Density	Average Negative Emotion Index	Correlation Coefficient	Significance Index
Excellent air	23.63	17.75	−0.432	0.615
Good air	55.6	16.00	0.285	0.531
Light haze	95.95	90.95	0.750	0.030
Moderate haze	124.67	28.00	0.281	0.518
Heavy haze	226.54	106.75	0.829	0.023

**Table 7:** Statistics and correlation analysis results of the four periods in November.

Periods	Average PM2.5 Density	Average Negative Emotion Index	Correlation Coefficient	Significance Index
02 November–07 November	49.67	23.17	0.614	0.195
12 November–17 November	51.50	9.50	0.229	0.662
18 November–23 November	181	57.33	0.887	0.018
25 November–30 November	155.33	59.00	0.735	0.096

## References

[1] Watts N., Adger W.N., Agnolucci P., Blackstock J., Costello P.A. (2015). Health and climate change: Policy responses to protect public health. Lancet.

[2] Lujala P., Lein H., Rød J.K. (2015). Climate change, natural hazards, and risk perception: The role of proximity and personal experience. Local Environ..

[3] Amann M., Klimont Z., Wagner F. (2013). Regional and Global Emissions of Air Pollutants: Recent Trends and Future Scenarios. Ann. Rev. Environ. Resour..

[4] Lin Y.C., Hsu S.C., Chou C.C.K., Zhang R., Wu Y., Kao S.J., Luo L., Huang C.H., Lin S.H., Huang Y.T. (2016). Wintertime haze deterioration in Beijing by industrial pollution deduced from trace metal fingerprints and enhanced health risk by heavy metals. Environ. Pollut..

[5] Cheng Z., Luo L., Wang S., Wang Y., Sharma S., Shimadera H., Wang X., Bressi M., de Miranda R.M., Jiang J. (2016). Status and characteristics of ambient PM2. 5 pollution in global megacities. Environ. Int..

[6] Zhang Y.L., Cao F. (2015). Fine particulate matter (PM 2.5) in China at a city level. Sci. Rep..

[7] Zhao W., Cheng J., Li D., Duan Y., Wei H., Ji R., Wang W. (2013). Urban ambient air quality investigation and health risk assessment during haze and non–haze periods in Shanghai, China. Atmos. Pollut. Res..

[8] Betha R., Behera S.N., Balasubramanian R. (2014). 2013 Southeast Asian smoke haze: Fractionation of particulate-bound elements and associated health risk. Environ. Sci. Technol..

[9] Xu P., Chen Y., Ye X. (2013). Haze, air pollution, and health in China. Lancet.

[10] Fujii Y., Tohno S., Amil N., Latif M.T., Oda M., Matsumoto J., Mizohata A. (2015). Annual variations of carbonaceous PM 2.5 in Malaysia: Influence by Indonesian peatland fires. Atmos. Chem. Phys..

[11] Gall E.T., Chen A., Chang V.W.C., Nazaroff W.W. (2015). Exposure to particulate matter and ozone of outdoor origin in Singapore. Build. Environ..

[12] Zhang J., Cui M., Fan D., Zhang D.S., Li J. (2015). Relationship between haze and acute cardiovascular, cerebrovascular, and respiratory diseases in Beijing. Environ. Sci. Pollut. Res..

[13] Zhang Z., Wang J., Chen L., Chen X., Sun G., Zhong N., Kan H., Lu W. (2014). Impact of haze and air pollution-related hazards on hospital admissions in Guangzhou, China. Environ. Sci. Pollut. Res..

[14] Fajersztajn L., Veras M., Barrozo L.V., Saldiva P. (2013). Air pollution: A potentially modifiable risk factor for lung cancer. Nat. Rev. Cancer.

[15] Isley C.F., Nelson P.F., Taylor M.P., Stelcer E., Atanacio A.J., Cohen D.D., Mani F.S., Maata M. (2018). Reducing mortality risk by targeting specific air pollution sources: Suva, Fiji. Sci. Total Environ..

[16] Li H., Wu H., Wang Q., Yang M., Li F., Sun Y., Qian X., Wang J., Wang C. (2017). Chemical partitioning of fine particle-bound metals on haze–fog and non-haze–fog days in Nanjing, China and its contribution to human health risks. Atmos. Res..

[17] Zheng G.J., Duan F.K., Su H., Ma Y.L., Cheng Y., Zheng B., Zhang Q., Huang T., Kimoto T., Chang D. (2015). Exploring the severe winter haze in Beijing: The impact of synoptic weather, regional transport and heterogeneous reactions. Atmos. Chem. Phys..

[18] Huang R.J., Zhang Y., Bozzetti C., Ho K.F., Cao J.J., Han Y., Daellenbach K.R., Slowik J.G., Platt S.M., Canonaco F. (2014). High secondary aerosol contribution to particulate pollution during haze events in China. Nature.

[19] Liu C., Hsu P.C., Lee H.W., Ye M., Zheng G., Liu N., Li W., Cui Y. (2015). Transparent air filter for high-efficiency PM 2.5 capture. Nat. Commun..

[20] Zhou J., Chen A., Cao Q., Yang B., Chang V.W.C., Nazaroff W.W. (2015). Particle exposure during the 2013 haze in Singapore: Importance of the built environment. Build. Environ..

[21] Wang L.T., Wei Z., Yang J., Zhang Y., Zhang F.F., Su J., Meng C.C., Zhang Q. (2014). The 2013 severe haze over southern Hebei, China: Model evaluation, source apportionment, and policy implications. Atmos. Chem. Phys..

[22] Gao J., Woodward A., Vardoulakis S., Kovats S., Wilkinson P., Li L., Xu L., Li J., Yang J., Li J. (2017). Haze, public health and mitigation measures in China: A review of the current evidence for further policy response. Sci. Total Environ..

[23] Huang L., Rao C., van der Kuijp T.J., Bi J., Liu Y. (2017). A comparison of individual exposure, perception, and acceptable levels of PM2. 5 with air pollution policy objectives in China. Environ. Res..

[24] Gifford R. (2014). Environmental psychology matters. Ann. Rev. Psychol..

[25] Fitzgerald D., Rose N., Singh I. (2016). Revitalizing sociology: Urban life and mental illness between history and the present. Br. J. Sociol..

[26] Triguero-Mas M., Dadvand P., Cirach M., Martínez D., Medina A., Mompart A., Basagaña X., Gražulevičienė R., Nieuwenhuijsen M.J. (2015). Natural outdoor environments and mental and physical health: Relationships and mechanisms. Environ. Int..

[27] Gascon M., Triguero-Mas M., Martínez D., Dadvand P., Forns J., Plasència A., Nieuwenhuijsen M.J. (2015). Mental health benefits of long-term exposure to residential green and blue spaces: A systematic review. Int. J. Environ. Res. Public Health.

[28] Ruijsbroek A., Mohnen S.M., Droomers M., Kruize H., Gidlow C., Gražulevičiene R., Andrusaityte S., Maas J., Nieuwenhuijsen M.J., Triguero-Mas M. (2017). Neighbourhood green space, social environment and mental health: An examination in four European cities. Int. J. Public Health.

[29] Spence A., Pidgeon N. (2009). Psychology, climate change & sustainable bahaviour. Environ. Sci. Policy Sustain. Dev..

[30] Bourque F., Cunsolo Willox A. (2014). Climate change: The next challenge for public mental health?. Int. Rev. Psychiatr..

[31] Berry H.L., Waite T.D., Dear K.B., Capon A.G., Murray V. (2018). The case for systems thinking about climate change and mental health. Nat. Clim. Chang..

[32] Dehaan E., Madsen J., Piotroski J.D. (2017). Do Weather-Induced Moods Affect the Processing of Earnings News?. J. Account. Res..

[33] Baylis P., Obradovich N., Kryvasheyeu Y., Chen H., Coviello L., Moro E., Cebrian M., Fowler J.H. (2018). Weather impacts expressed sentiment. PLoS ONE.

[34] Denissen J.J., Butalid L., Penke L., Van Aken M.A. (2008). The effects of weather on daily mood: A multilevel approach. Emotion.

[35] Kööts L., Realo A., Allik J. (2011). The influence of the weather on affective experience: An experience sampling study. J. Individ. Differ..

[36] Lucas R.E., Lawless N.M. (2013). Does life seem better on a sunny day? Examining the association between daily weather conditions and life satisfaction judgments. J. Personal. Soc. Psychol..

[37] Zhang X., Zhang X., Chen X. (2017). Happiness in the air: How does a dirty sky affect mental health and subjective well-being?. J. Environ. Econ. Manag..

[38] Kaplan A.M., Haenlein M. (2010). Users of the world, unite! The challenges and opportunities of Social Media. Bus. Horiz..

[39] Qiu L., Lin H., Ramsay J., Yang F. (2012). You are what you tweet: Personality expression and perception on Twitter. J. Res. Personal..

[40] Stieglitz S., Dang-Xuan L. (2013). emotion and information diffusion in social media—sentiment of microblogs and sharing behavior. J. Manag. Inf. Syst..

[41] Abdul-Mageed M., Diab M., Kübler S. (2014). SAMAR: Subjectivity and sentiment analysis for Arabic social media. Comput. Speech Lang..

[42] Cao N., Lu L., Lin Y.R., Wang F., Wen Z. (2015). Socialhelix: Visual analysis of sentiment divergence in social media. J. Visual..

[43] Ceron A., Curini L., Iacus S.M., Porro G. (2014). Every tweet counts? How sentiment analysis of social media can improve our knowledge of citizens’ political preferences with an application to Italy and France. New Media Soc..

[44] Nguyen T.H., Shirai K., Velcin J. (2015). Sentiment analysis on social media for stock movement prediction. Expert Syst. Appl..

[45] Zheludev I., Smith R., Aste T. (2014). When can social media lead financial markets?. Sci. Rep..

[46] Burnap P., Williams M.L., Sloan L., Rana O., Housley W., Edwards A., Knight V., Procter R., Voss A. (2014). Tweeting the terror: Modelling the social media reaction to the Woolwich terrorist attack. Soc. Netw. Anal. Min..

[47] Gaspar R., Pedro C., Panagiotopoulos P., Seibt B. (2016). Beyond positive or negative: Qualitative sentiment analysis of social media reactions to unexpected stressful events. Comput. Hum. Behav..

[48] Sina microblog. https://weibo.com/.

[49] Nakov P., Rosenthal S., Kiritchenko S., Mohammad S.M., Kozareva Z., Ritter A., Stoyanov V., Zhu X. (2016). Developing a successful SemEval task in sentiment analysis of Twitter and other social media texts. Lang. Resour. Eval..

[50] Villarroel Ordenes F., Ludwig S., De Ruyter K., Grewal D., Wetzels M. (2017). Unveiling what is written in the stars: Analyzing explicit, implicit, and discourse patterns of sentiment in social media. J. Consum. Res..

[51] Rout J.K., Choo K.K., Dash A.K., Bakshi S., Jena S.K., Williams K.L. (2018). A model for sentiment and emotion analysis of unstructured social media text. Electron. Comm. Res..

[52] Pepe A., Bollen J. Between conjecture and memento: Shaping a collective emotional perception of the future. Proceedings of the AAAI Spring Symposiumon Emotion, Personality, and Social Behavior.

[53] Bollen J., Mao H.M., Zeng X.J. (2011). Twitter mood predicts the stock market. J. Comput. Sci..

[54] Zhao J., Dong L., Wu J., Xu K. MoodLens: An emoticon-based sentiment analysis system for chinese tweets. Proceedings of the 18th ACM SIGKDD International Conference on Knowledge Discovery and Data Mining.

[55] Quan C., Ren F. (2010). A blog emotion corpus for emotional expression analysis in Chinese. Comput. Speech Lang..

[56] NLPIR-ICTCLAS. http://ictclas.nlpir.org/.

[57] Watson D., Tellegen A. (1985). Toward a consensual structure of mood. Psychol. Bull..

